# Contribution of Tau Pathology to Mitochondrial Impairment in Neurodegeneration

**DOI:** 10.3389/fnins.2018.00441

**Published:** 2018-07-05

**Authors:** María J. Pérez, Claudia Jara, Rodrigo A. Quintanilla

**Affiliations:** ^1^Laboratory of Neurodegenerative Diseases, Universidad Autónoma de Chile, Santiago, Chile; ^2^Centro de Investigación y Estudio del Consumo de Alcohol en Adolescentes (CIAA), Santiago, Chile

**Keywords:** tau, mitochondria, Alzheimer’s disease, synapse neurodegeneration, synapsis

## Abstract

Tau is an essential protein that physiologically promotes the assembly and stabilization of microtubules, and participates in neuronal development, axonal transport, and neuronal polarity. However, in a number of neurodegenerative diseases, including Alzheimer’s disease (AD), tau undergoes pathological modifications in which soluble tau assembles into insoluble filaments, leading to synaptic failure and neurodegeneration. Mitochondria are responsible for energy supply, detoxification, and communication in brain cells, and important evidence suggests that mitochondrial failure could have a pivotal role in the pathogenesis of AD. In this context, our group and others investigated the negative effects of tau pathology on specific neuronal functions. In particular, we observed that the presence of these tau forms could affect mitochondrial function at three different levels: (i) mitochondrial transport, (ii) morphology, and (iii) bioenergetics. Therefore, mitochondrial dysfunction mediated by anomalous tau modifications represents a novel mechanism by which these forms contribute to the pathogenesis of AD. In this review, we will discuss the main results reported on pathological tau modifications and their effects on mitochondrial function and their importance for the synaptic communication and neurodegeneration.

## Introduction

Tau protein, which belongs to the family of microtubule-associated proteins (MAPs), was first discovered in 1975 and identified as a molecule that physiologically promotes the assembly and stabilization of microtubules (Weingarten et al., 1975). Tau is mainly expressed in neurons and is present in great extent in axons controlling neuronal development (Ding et al., 2006; McMillan et al., 2011; Kolarova et al., 2012), promoting the vesicular and axonal transport (Dolan and Johnson, 2010; Rodriguez-Martin et al., 2013) and is critical in defining the polarity of neurons (Avila et al., 2016). In a number of human diseases called tauopathies, including Progressive Supranuclear Palsy (PSP), Pick’s disease (PiD), Down’s syndrome (DS), and Frontotemporal Dementia and Parkinsonism linked to chromosome 17 (FTDP-17), soluble tau assembles into insoluble filaments, leading to synaptic failure and neurodegeneration (Spillantini and Goedert, 2013). Moreover, in AD, one of the most common forms of dementia in elderly, tau undergoes specific pathological modifications that are the principal components of neurofibrillary tangles (NFTs), one of the main neuropathological hallmarks of AD (Kosik et al., 1986).

**FIGURE 1:**
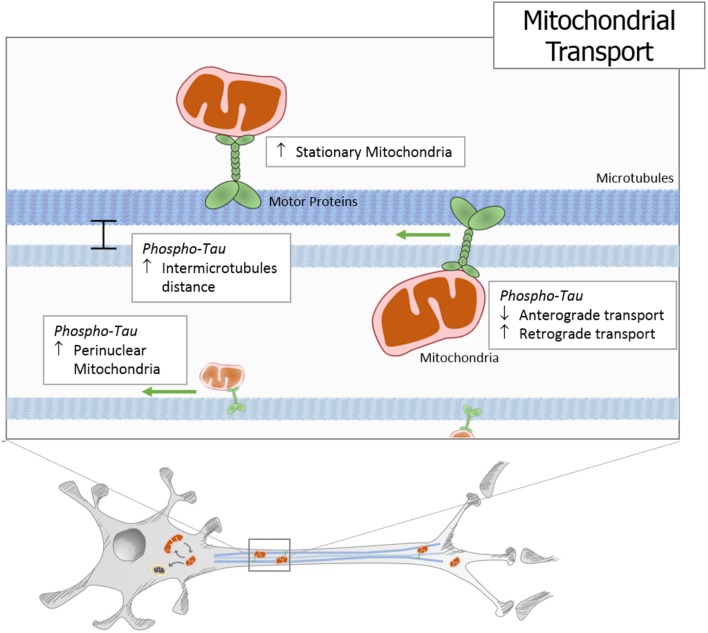
Tau pathology impairs mitochondrial transport. In different cells and animal models, it has been described that hyperphosphorylated and truncated tau generate an increase in stationary mitochondria. Moreover, studies using different tau pathology models show an increase in the intermicrotubular distance that could be responsible for the mitochondrial movement reduction. At the same time, neuronal models that express pathological forms of tau show a decrease in anterograde transport and an increase in retrograde transport, which finally leads to an increase in perinuclear mitochondria.

**FIGURE 2:**
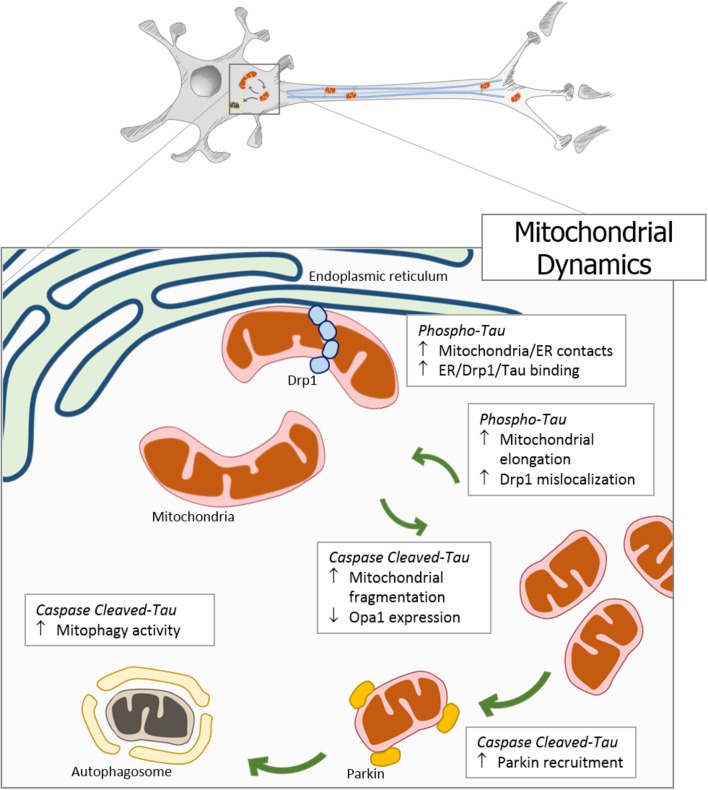
Pathological forms of tau affect mitochondrial dynamics. In neurodegenerative diseases, the accumulation of pathological forms of tau (hyperphosphorylated and cleaved) impairs the regulation of mitochondrial dynamics. The overexpression of phosphorylated tau generates an increase in mitochondrial length, a decrease in fission proteins, and an increase in DRP1 mislocalization. Interestingly, tau phosphorylation increases endoplasmic reticulum-mitochondria contacts promoting a close interaction between tau, DRP1, and ER. On the other hand, C-terminal caspase-cleaved tau induces mitochondrial fragmentation through the reduction of the Opa1 expression. In addition, the presence of truncated tau at N-end increases the Parkin recruitment to the mitochondria, triggering an inappropriate and excessive autophagy.

**FIGURE 3:**
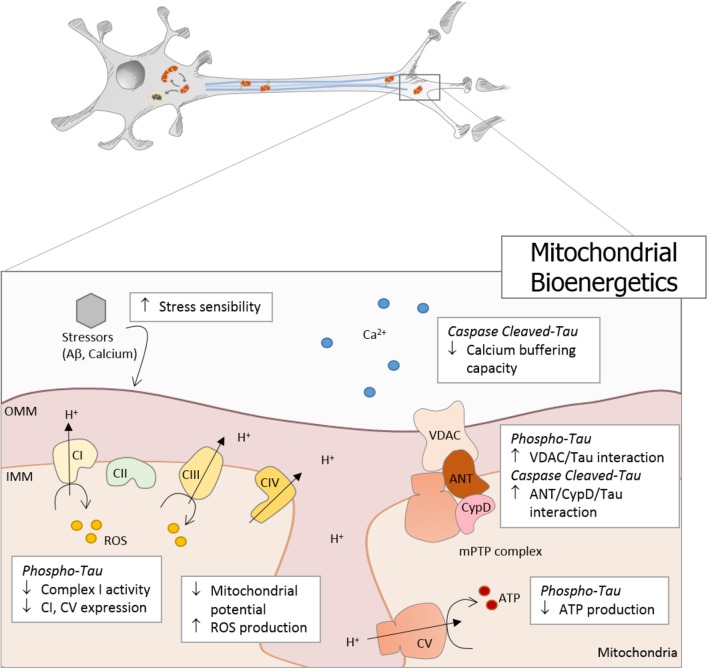
Tau pathology affects mitochondrial bioenergetics in AD. Mitochondrial bioenergetics is significantly affected by tau pathology in AD. Phosphorylated and truncated tau severely affects mitochondrial membrane potential (MMP) and induces oxidative stress, which finally increases the mitochondrial sensibility to different stressors, such as Aβ and calcium. Moreover, phosphorylated tau decreases the expression of complex I and V, with a concomitant reduction in complex I activity and ATP production. In addition, caspase-cleaved tau impairs mitochondrial function affecting the calcium buffering capacity. Both pathological forms of tau present dysfunctional characteristics that suggest the involvement of the mitochondrial permeability transition pore (mPTP) in the bioenergetics failure. Phosphorylated tau induces an increase in VDAC/Tau interaction, and N-end truncated tau promotes an increase in ANT/CypD/Tau binding.

Mitochondria are responsible for energy supply, detoxification, and communication in brain cells, and several investigations suggest that mitochondria failure could have a role in the pathogenesis of Parkinson’s disease, Huntington disease, and AD (Cabezas-Opazo et al., 2015). On the other hand, several studies suggest that mitochondrial dysfunction is an early event in the pathogenesis of AD and is involved in other tauopathies (Gibson and Shi, 2010; Cabezas-Opazo et al., 2015). In particular, the presence or accumulation of tau pathology can affect mitochondrial function at three specific points: (i) mitochondrial transport (**Figure 1**), (ii) dynamics (morphology) (**Figure 2**), and (iii) bioenergetics (**Figure 3**). Therefore, in this review, we discuss the major results reported on tau pathological modifications and their effects on mitochondrial function and its implications for the pathogenesis of AD.

## Tau Protein in Health and Disease

In the human genome, tau is encoded by a single gene, which is located on the long arm of chromosome 17 (Neve et al., 1986; Avila et al., 2004, 2016; Kolarova et al., 2012; Medina and Avila, 2014). When tau is routinely studied, it is often treated as if it was a single protein, whereas, in fact, it exists in six protein isoforms and several phosphorylated species whose functions are not entirely understood (McMillan et al., 2011; Andreadis, 2012; Kolarova et al., 2012; Medina and Avila, 2014; Derisbourg et al., 2015). The tau gene contains 16 exons and the different isoforms arise from the alternative splicing of exons 2, 3, and 10. Exons 2 and 3 are alternatively spliced, and adult human tau can contain exon 2 (one N-terminal insert or 1N), exons 2 and 3 (2N), or neither (0N) (Avila et al., 2004; Crespo-Biel et al., 2012; Kolarova et al., 2012; Medina and Avila, 2014). The N-terminal is projected from the microtubule surface, where it is believed to interact with cytoskeletal elements and the plasma membrane (Gong et al., 2005). The remainder protein contains the four microtubule-binding domains, encoded from exons 9, 10, 11, and 12. From these, only exon 10 is alternatively spliced and can either be spliced in (4R) or out (3R) (Goedert and Crowther, 1989; Goedert et al., 1989; Ittner et al., 2010; Martin et al., 2011; Kolarova et al., 2012; Pedersen and Sigurdsson, 2015). In addition, the C-terminal region also contains projection domains, suggesting that the different tau species must interact with specific subsets of proteins and execute specific cellular functions (Kolarova et al., 2012; Kadavath et al., 2015).

In physiological conditions, tau could be phosphorylated or dephosphorylated depending on the equilibrium between the actions of kinases (such as CDK5/p35 and GSK3β) and phosphatases (including PP1, PP2A, PP2B, and PP2C), respectively (Arrasate et al., 2000; Maas et al., 2000; Maccioni et al., 2001; Kolarova et al., 2012; Hernandez et al., 2013). This balance in the phosphorylation state of tau determines binding and stability of tubulin polymerization in neuronal cells, which is necessary for maintaining the structure of the axon and dendrites (Wolfe, 2012; Liu S.L. et al., 2016). In several neurodegenerative disorders, tau becomes hyperphosphorylated which alters its secondary structure and leads to conformational and functional alterations (Hanger and Wray, 2010). The loss of the binding ability of tau to microtubules due to hyperphosphorylation also facilitates the formation of paired helical filaments (PHFs), which leads to NFT formation in AD (de Calignon et al., 2010; Crespo-Biel et al., 2012). Those alterations cause destabilization of the cytoskeleton network and disruption of axonal transport (Alonso et al., 2001; Kolarova et al., 2012), which can also lead to irreversible changes in microtubule dynamics, neuronal dysfunction, synaptic damage, and ultimately cell death (Liu et al., 2007; Kolarova et al., 2012; Dorostkar et al., 2015).

Although phosphorylation is usually considered as one of the most important modifications of tau in AD, evidence suggests that the proteolysis impairment may play a significant role as an early step in neurodegeneration (Johnson, 2006). Tau protein is a substrate of several endogenous proteases (Wang et al., 2010), and among them, caspases and calpain have been investigated most intensively (Wang et al., 2010). Studies in cellular and animal models show that proteolysis processing of tau increases its propensity for aggregation, an event that was found to be relevant in the formation of NFTs which are composed of insoluble PHFs and accumulate inside neurons (Hanger et al., 2009). At the same time, caspase activation is related to the toxic effects mediated by amyloid β-peptide (Aβ) in AD (Rissman et al., 2004; Hanger and Wray, 2010). In that context, it has been found that tau can be cleaved at several residues of the carboxy-terminal region (Gamblin et al., 2003), preferentially by caspase-3 cleaving at aspartic acid 421 (Asp 421) (Ding et al., 2006; Quintanilla et al., 2012). On the other hand, the digestion of native PHFs leaves a filament fragment that contains only a 12 kDa tau protein truncated at glutamic acid 391 (Glu391) (Garcia-Sierra et al., 2008). This C-terminal proteolytic process induces self-aggregation and increases the rate of polymerization of tau, which later promotes formation and assembly of NFTs (Gamblin et al., 2003; Ding et al., 2006; de Calignon et al., 2010; Hanger and Wray, 2010; Kolarova et al., 2012), which leads to dendritic spine loss, synaptic impairment, and memory deficits (D’Amelio et al., 2011; Zhao et al., 2015). Another truncation of tau occurs in the N-terminal region by the action of caspase-6 (Wang et al., 2010; Kolarova et al., 2012). N-terminal tau fragments have been found in several *in vitro* and *in vivo* models, such as in primary neuronal cultures undergoing apoptosis (Canu et al., 1998; Park and Ferreira, 2005), in the cerebrospinal fluid (CSF) of rats after traumatic brain injury (TBI), in transient forebrain ischemia (Siman et al., 2004), and in brain tissue of AD patients (Rohn et al., 2002). Further reports have demonstrated that a significant proportion of 20–22 kDa N-terminal tau fragments (NH2hTau) is preferentially located in the mitochondria-rich synapses from AD hippocampus and frontal cortex. In addition, this NH2hTau fragment is associated with neurofibrillary degeneration and synaptic impairment in human AD brains (Amadoro et al., 2010). Although this is not an early event in AD, these findings suggest that N-terminal tau truncation contributes to the progression of the disease and is a critical step in the toxic cascade leading to neuronal death, similar to what has been proposed for the C-terminal cleavage of tau by caspases (Fasulo et al., 2000, 2005).

It is clear that even under normal physiological conditions tau may undergo different posttranslational modifications, such as phosphorylation, acetylation, glycation, ubiquitination, nitration, truncations (proteolytic cleavage), and abnormal conformational changes (Hanger and Wray, 2010; Pritchard et al., 2011; Kolarova et al., 2012; Kumar et al., 2014; Tenreiro et al., 2014). To this date, it is unknown when and how these posttranslational modifications affect tau functions and triggering different pathological conditions (Bodea et al., 2016). However, these abnormal tau conformations generate serious alterations in neuronal activity, causing a loss in its ability to transmit synaptic signals, and contribute to dendritic spine loss (Dorostkar et al., 2015). Interestingly, in the last years, it was hypothesized that abnormalities in tau function may also accelerate the development of several signs of neurotoxicity or become neurons more vulnerable to insults, which includes oxidative stress, calcium dysregulation, inflammation, mitochondrial impairment, and excitotoxicity (Gendron and Petrucelli, 2009). This suggests a direct participation of tau as an intermediary in these processes.

In that context, several studies using tau knockout (KO) mice have shown a protection from neurotoxicity induced by Aβ treatment compared to wild-type (WT) mice (Rapoport et al., 2002; Roberson et al., 2007). Furthermore, Roberson and collaborators described that reducing the endogenous tau levels prevented behavioral deficits caused by Aβ and protected against excitotoxicity (Roberson et al., 2007). The morphological analysis shows that WT neurons degenerate in the presence of Aβ, while tau-depleted neurons show no signs of degeneration in those conditions (Rapoport et al., 2002). In a similar fashion, blocking tau expression with an antisense oligonucleotide completely blocks Aβ toxicity in differentiated primary neurons (Liu et al., 2004). These results provide direct evidence supporting a key role for tau in the mechanisms leading to Aβ-induced neurodegeneration in the central nervous system (Gendron and Petrucelli, 2009) and predict that only cells containing appreciable levels of tau are susceptible to Aβ toxicity (Rapoport et al., 2002). On the other hand, it was described that tau KO mice are not only protected from Aβ neurotoxicity but also against the effects of neurologic stress. For example, Lopes and colleagues describe that the reduction of tau expression protects from working memory impairments, dendritic spine loss, and synaptic failure induced in the prefrontal cortex (PFC) of a chronic stress mouse model (Lopes et al., 2016). Interestingly, this study suggests that stress-induced neuronal damage and cognitive decline depend on an interaction between tau and several mitochondrial proteins that affects mitochondrial localization at the synapses. Therefore, it is highly plausible that the ablation of tau expression prevents mitochondria motility impairment leading to a protection of dendrites and synapses against stress (Lopes et al., 2016).

Interestingly, the relationship between the reduction of tau expression and the improvement of mitochondrial health has been previously suggested (Vossel et al., 2010). Vossel and collaborators describe that neurons from WT animals present an impaired axonal motility of mitochondria in the presence of Aβ, an effect that was stronger for anterograde than retrograde transport. However, the complete or partial reduction of tau expression prevents these defects without affecting the axonal transport baseline (Vossel et al., 2010). Other groups describe that neurons from tau KO mice are also resistant to Aβ-induced mitochondrial damage compared to neurons obtained from WT mice (Pallo and Johnson, 2015). However, tau KO neurons show a more pronounced cytosolic calcium elevation in response to Aβ, suggesting that tau may facilitate Aβ-induced mitochondrial damage in a manner that is independent of cytosolic calcium increase (Pallo and Johnson, 2015). Complementary studies show that tau protein interacts either directly or indirectly with more than 100 proteins in physiological conditions. Almost half of them were grouped as membrane-bound proteins, and mitochondrial proteins composed the largest fraction (40.4%) of this group. In addition, this study identified several proteins with a preferential binding to distinct isoforms of tau, and it was described that the specific 2N tau isoforms have preferential binding to proteins involved in ATP biosynthesis and synaptic transmission. Interestingly, these proteins are abundant in several neurodegenerative diseases, dementia, and tauopathies, suggesting a tight relationship between tau, mitochondria, and synaptic transmission (Liu C. et al., 2016).

Regarding the relationship between mitochondria and the neuronal damage, it seems logical to think that defects in mitochondria trafficking could result in a decrease in local ATP levels which could impair synapses (Gendron and Petrucelli, 2009). Also, mitochondrial dysfunction can generate an imbalance in the calcium buffering capacity and the consequent release of presynaptic glutamate or impair the clearance of glutamate from the synapse, thus leading to high levels of extracellular glutamate thereby inducing neurotoxicity (Di Monte et al., 1999; Bobba et al., 2004). In this context, it is necessary to investigate the relationship between tau protein, its pathological modifications, mitochondrial function, and neuronal damage.

## Mitochondrial Transport and Tau Pathology

As we already mentioned, microtubule-associated proteins such as MAPs regulate the organization, polymerization, and stability of microtubules. Tau localizes predominantly in the axons where it participates in the regulation of the stability and assembly of microtubules (Gendron and Petrucelli, 2009). Microtubules, one of the principal components of the cytoskeleton, are involved in the maintenance of neuronal morphology and the formation of axonal and dendritic processes, and they play a vital role in cellular trafficking (Cabezas-Opazo et al., 2015). The transport of cargo proteins to different parts of neurons is critical for their synaptic functions, as motor proteins like kinesin and dynein travel along the microtubules to and from pre- and postsynaptic sites. These actions allow the movement of mitochondria, synaptic vesicles, ion channels, receptors, and scaffolding proteins through neurons (Hollenbeck and Saxton, 2005; Bereiter-Hahn and Jendrach, 2010). Synapses are highly vulnerable to the impairment in microtubule transport; therefore, any perturbation in this communication system could cause neurotransmission impairment and lead to synaptic degeneration (Gendron and Petrucelli, 2009).

It has been proposed, that pathological forms of tau could disrupt axonal transport and cause synaptic damage by several mechanisms. Tau overexpression increases the pausing frequency of mitochondria movement by 16% in neurons (Shahpasand et al., 2012). In addition, overexpression of tau reduces anterograde movement of mitochondria, suggesting that tau itself inhibits mitochondrial transport independent of its posttranscriptional modification state (Shahpasand et al., 2012). Therefore, this indicates that overload of tau binding to microtubules results in a transport inhibition essentially by blocking the movement of the motor proteins (Stamer et al., 2002).

Overexpression of tau modifications can also destabilize microtubules leading to microtubule disassembly, thus impairing the microtubule tracks needed for the transport of molecular motors and their cargo (Alonso et al., 1994). In an *in vitro* model of overexpression of pathological tau forms, it was shown that tau hyperphosphorylated at the AT8 sites (Ser199/Ser202/Thr205) inhibits mitochondrial transport to a greater degree than WT tau by increasing the inter-microtubule distance (Shahpasand et al., 2012). On the other hand, SH-SY5Y cells stably overexpressing either human WT tau or the tau carrying the P301L FTDP-17 mutation present a significant decrease in mitochondrial movement, causing a destabilization of the microtubule network, which leads to a perinuclear localization of the mitochondria (Schulz et al., 2012). In addition, in rTg4510 mice, a mouse model that overexpresses the P301L mutation and develops a robust NFT-like pathology at 4–5 months of age, an aberrant mitochondrial distribution is evident in neurites regardless of whether or not aggregated tau is present in the form of neutropil threads (Kopeikina et al., 2011). The somatic distribution is less affected, as only cells with somatic accumulation of misfolded tau show indications of perinuclear mitochondrial clumping, and this phenotype is worsening with age (Kopeikina et al., 2011). Interestingly, this work suggests tau-induced anterograde transport deficits as the general mechanism by which mitochondria become improperly distributed (Kopeikina et al., 2011). Therefore, it is possible that the ability of tau to impair axonal transport does not necessarily involve microtubule dysfunction. That is because, in most models, retrograde transport is not impaired, and for that, it seems unlikely that the inhibition of anterograde axonal transport resulting from tau overexpression is caused only by altered microtubule dynamics (Gendron and Petrucelli, 2009). The alternative is that tau itself could be interacting with kinesin (Utton et al., 2005; Cuchillo-Ibanez et al., 2008; Dubey et al., 2008), raising the possibility that high levels of tau may compete with potential kinesin cargo and thus prevent their translocation to the synapse.

Furthermore, in cortical neurons from the knockin (KI) P301L mice, expression of mutated tau at physiological levels also suggests mitochondrial transport defects (Rodriguez-Martin et al., 2016). In this model, a reduced number of axonal mitochondria was found, without differences in kinetic parameters of anterograde and retrograde transport (Rodriguez-Martin et al., 2016). Furthermore, neurons that were expressing the KI-P301L tau mutation showed a negative alteration in the angle that defines the orientation of the mitochondria in the axon. Such change in the orientation could lead to a temporary blockage of axonal mitochondria that could increase the mitochondrial fusion and decrease the number of mitochondria in the axon (Rodriguez-Martin et al., 2016). On the other hand, tau could also be involved in the trafficking deficits elicited by MDMA (commonly known as ecstasy), as confirmed by the partial reversion of mitochondrial movement deficits in tau KO neurons treated with this drug (Barbosa et al., 2014). MDMA treatment increases tau hyperphosphorylation mediated by GSK3β, and the blocking of this activity reduces MDMA-induced mitochondrial trafficking alterations (Barbosa et al., 2014). These results together suggest that physiological levels of modified tau protein could also alter mitochondrial transport.

Interestingly, other work has shown that an increase in phosphorylated tau is correlated with an increase in mitochondrial movement from cytoplasm to synapses in hippocampal neurons of a chronic stress mouse model (Zhang et al., 2012). Consistent with the *in vivo* model, the mitochondrial transport was decreased in cultured primary hippocampal neurons when hyperphosphorylated tau was inhibited by lithium (Zhang et al., 2012). On the other hand, overexpression of GSK3β in cultured neurons results in an increase in the number of motile axonal mitochondria (Llorens-Martin et al., 2011). This effect was completely abolished in tau KO mice, indicating that the effects of this kinase are mediated by its action on tau, as an overexpression of GSK3β results in an increase in tau hyperphosphorylation and a decrease of tau binding to microtubules (Llorens-Martin et al., 2011). These different approaches have shown that an increase in phosphorylated tau is not always related with a decrease in mitochondrial movement. However, it is a fact that changes in tau levels and their posttranslational modifications directly influence the mitochondrial transport in neurons and finally affect the synaptic process (**Figure 1**).

## Effects of Tau Pathology on Mitochondrial Dynamics and Mitophagy

Mitochondria are the powerhouse of the cell. Apart from the energy production, they play important roles in many cellular activities, such as metabolism, aging, and cell death (Nunnari and Suomalainen, 2012). Because of the high demand of energy and the characteristics mitochondrial metabolism, neurons contain through the cytoplasm and axons many mitochondria are maintained as short tubular structures with high dynamic actions (Kageyama et al., 2011). Fission and fusion proteins, that regulate organelle size, number, and shape and contribute to the correct function of the organelle, coordinate the dynamic interactions among mitochondria (Itoh et al., 2013). The soluble cytosolic protein that assembles into spiral filaments around mitochondrial tubules, dynamin-related GTPase (Drp1), mediates mitochondrial fission (Kageyama et al., 2011; Tamura et al., 2011). Drp1 regulates its interactions with mitochondria trough several posttranslational modifications, including phosphorylation, ubiquitination, and sumoylation (Chang and Blackstone, 2010). Also, this protein can interact with other outer membrane proteins, including Mff, Fis1, and the two homologous proteins Mid49 and Mid51, that contribute to the mitochondrial division (Kageyama et al., 2011). On the other hand, mitochondrial fusion is mediated by the dynamin-related GTPases mitofusin 1 and 2 (Mfn1/2) and optic dominant atrophy 1 (Opa1) (Tamura et al., 2011; Wilson et al., 2013). Mfns are located in the outer membrane and are subjected to ubiquitination and proteasomal degradation (Gegg et al., 2010; Tanaka, 2010), while Opa1 is located in the inner mitochondrial membrane where it is proteolytically regulated by different mitochondrial proteases (Ishihara et al., 2006; Quiros et al., 2012). Opa1 and Mfns interact to form mitochondrial intermembrane complexes that promote the fusion of outer and inner mitochondrial membranes (Song et al., 2009). An imbalance in one of these proteins leads to a similar mitochondrial fragmentation phenotype, suggesting that both outer and inner membrane fusion processes are affected (Itoh et al., 2013).

Many age-related neurodegenerative diseases are associated with alterations in the fission and fusion of mitochondria (Cho et al., 2010). For example, in brain studies of higher order animals it was reported important changes in mitochondrial morphology from aged rhesus macaque (RM), including numerous mitochondria with different size profiles, related with an unfinished fission by Drp1 and Fis1 proteins (Morozov et al., 2017). Despite that changes in RM brain were most similar to healthy elderly humans than AD pathology (Cramer et al., 2018); those studies suggest that alterations in mitochondrial morphology in normal brain aging may contribute to cognitive decline. Remains to be determinate the contribution of tau protein in those processes.

Overexpression of human tau (hTau) in different cell types (HEK 293, primary neurons, and neuronal cultures from hTau transgenic mice) not only enhances retrograde mitochondrial transport rate but also mitochondrial fusion, which may explain the perinuclear mitochondrial accumulation. The fusion proteins Mfns and Opa1 are significantly increased and fission proteins are not changed (Li et al., 2016). Reduced polyubiquitinated Mfn2 was also found, suggesting that an impaired ubiquitination may underlie Mfn accumulation. Interestingly, in the same studies, the downregulation of Mfn2 prevented the hTau-induced mitochondrial injury and the cell viability loss (Li et al., 2016). Related with this specific effect, mitochondria from WT tau expressing cells show an orthodox mitochondrial state with small intracristal spaces, contrary to the globular structure of cristae and dense matrix that is present in mitochondria of cells bearing the P301L mutation (Li et al., 2016). Furthermore, the P301L tau mutation leads to an impairment of mitochondrial fission and fusion generated by a reduced expression of the fusion factors Mfn1 and Opa1 and all fission factors (Schulz et al., 2012).

Complementary studies show that expression of hTau results in the elongation of mitochondria in both Drosophila and mouse neurons, an event that is enhanced by the expression of tau R406W (a human mutation that enhances toxicity in the aging brain) (DuBoff et al., 2012). In addition, a greater mitochondrial elongation was triggered by expression of a more toxic, pseudo-hyperphosphorylated form of tau (tau E14) (DuBoff et al., 2012). This event seems to be related with a mislocalization of Drp1 with a subsequent failure of normal mitochondria dynamics control. In addition, mitochondrial elongation is accompanied by the excessive production of reactive oxygen species (ROS) and cell cycle-mediated death, which can be rescued *in vivo* by genetically restoring the proper balance of mitochondrial fission and fusion (DuBoff et al., 2012). More important, in postmortem brain of AD patients and brain tissues from APP, APP/PS1, and 3xTgAD mice, it was found that phosphorylated tau interacts with Drp1 and that this interaction occurs mainly at a late stage of disease progression (Manczak and Reddy, 2012a). Those effects are accompanied by an increase in GTPase activity and it appears that the interaction between Drp1 and hyperphosphorylated tau exacerbates mitochondrial and synaptic deficiencies, ultimately leading to neuronal damage and cognitive decline in AD (Manczak and Reddy, 2012a). Interestingly, it has been suggested that prior to the assembly of Drp1 filaments into the mitochondria the endoplasmic reticulum (ER) wraps around the organelle at an early stage of division (Friedman et al., 2011). These ER-mitochondria contacts may help Drp1 to assemble and, after fission of the mitochondria, Drp1 to disassemble from ER-mitochondria contacts for future rounds of mitochondrial fission (Friedman et al., 2011). In motor neurons of JNPL3 mice overexpressing tau P301L, an increase in the numerous contacts between ER and mitochondria compared to WT mice was shown (Friedman et al., 2011). In addition, tau immunogold labeling indicates that this increased number of contacts might result from the preferential association of tau with ER membranes. Interestingly, this association pattern was shown in AD brains and indicates an imbalance of mitochondrial fission mediated by Drp1 (Perreault et al., 2009).

It is important to remember that phosphorylation is not the only important modification of tau as it was suggested that tau truncation could be an early step in neurodegeneration (Johnson, 2006). Results from our group show that expression of caspase-cleaved tau in a neuronal cell model results in mitochondrial impairment (Quintanilla et al., 2012, 2014). We have shown that truncated tau alone induces an increase in mitochondrial fragmentation in neurons. In addition, when transfected cells are treated with Aβ at sublethal concentrations, there is an increase in the stationary mitochondrial population (Quintanilla et al., 2012, 2014). Also, we have recently described that this impairment in mitochondrial morphology is mediated by a decrease in Opa1 levels in neuronal cells (Perez et al., 2017). These effects are likely to affect mitochondrial bioenergetics and neuronal function since the presence of truncated tau enhances mitochondrial damage and cell viability loss induced by Aβ (Perez et al., 2017).

Interestingly, it has been widely described that the regulation of mitochondrial morphology by fission is important for the clearance of this organelle by mitophagy (Shutt et al., 2011). During this autophagy-mediated degradation of mitochondria, mitochondrial fission is enhanced partly due to the proteasomal degradation of Mfns and the proteolytic processing of Opa1 (Gegg et al., 2010; Tanaka, 2010; Ziviani and Whitworth, 2010). Whereas an increase in mitochondrial division facilitates the mitophagy of dysfunctional mitochondria, fission is downregulated by a Drp1 phosphorylation mechanism during starvation-induced autophagy, resulting in elongated mitochondrial networks that are protected against degradation (Itoh et al., 2013). After the fragmentation process, Parkin, an E3 ubiquitin ligase, is recruited to dysfunctional mitochondria and ubiquitinates mitochondrial proteins for proteasomal degradation and promotes the engulfment of mitochondria by autophagosomes (Shutt et al., 2011).

In that context, intracellular accumulation of WT hTau results in mitophagy deficits, as the increase in hTau may block the transport of autophagosomes (Hu et al., 2016). In addition, Parkin levels are reduced in the mitochondrial fraction of hTau transfected cells, and tau directly inserts into the outer membrane fraction, which could be reduce the interaction between Parkin and mitochondria (Hu et al., 2016). Taken together these events suggest that overexpression of hTau itself generates a massive accumulation of dysfunctional mitochondria in somatodendritic compartments of neurons. In the case of cleaved tau, specifically the NH2hTau fragment, an impairment in its selective autophagic clearance and the mitochondrial dynamics was described. Fragmentation and perinuclear mislocalization of mitochondria with smaller size and density are early found in dying NH2hTau-expressing neurons (Amadoro et al., 2014). This effect could be related with a reduction in the general Parkin-mediated remodeling of the proteosome membrane, an increase in the colocalization of mitochondria with autophagic markers, bioenergetics deficits, and *in vitro* synaptic pathology (Amadoro et al., 2014). Interestingly, a later work by the same group shows that NH2hTau generates an imbalance in Parkin-mediated mitophagy favoring cell death in a neuronal model. The NH2hTau fragment modifies the quality control of neuronal mitochondria by facilitating subcellular trafficking and/or recruitment of both Parkin and UCHL-1 to these organelles compelling them to inappropriate, excessive, and deleterious elimination via selective autophagy. Moreover, inhibition of excessive mitophagy in NH2hTau neurons partially restores the mitochondrial content but does not completely prevent the mitochondrial damage resulting in a modest but significant protection against cell death (Corsetti et al., 2015). All this evidence suggests that mitochondrial fragmentation, mitophagy, and neuronal death represent a mechanism of response to a mitochondrial bioenergetics damage generated by an overexpression of tau and/or an increase in its pathological processing (**Figure 2**).

## Mitochondrial Bioenergetics Failure and Tau Pathology

The main function of mitochondria is to convert the energy derived from nutrients into heat and ATP, but it is also a major contributor to calcium regulation, ROS production, cell metabolism, and cell death (Nunnari and Suomalainen, 2012). Under normal conditions, mitochondria can buffer substantial amounts of calcium during neurotransmission, and finely control oxidative stress in the brain (Friberg et al., 2002). In pathological conditions, mitochondrial bioenergetics dysfunction can occur, leading to neuronal degeneration and cell death (Mattson et al., 2008). In that context, it is currently controversial if overexpression of human tau produces mitochondrial damage in neuronal cell lines. Li et al. (2016) describe that hTau overexpression in primary culture decreases ATP levels and the ratio ATP/ADP as well as inhibits complex I activity. On the other hand, overexpression of WT tau in SH-SY5Y cells improves mitochondrial function through an increase in complex I activity, resulting in a hyperpolarized mitochondrial membrane potential (MMP), higher ATP levels, and an increased metabolic activity (Schulz et al., 2012). The exact reasons for the discrepancy are currently not clear, but the different cell types and transfection protocols may be one of them.

Despite that, it seems to be a common conclusion that overexpression of the P301L tau mutation leads to mitochondrial dysfunction. In neuronal models, a reduction in ATP levels, a slight depolarization of the MMP as well as decreased metabolic activity induced by a pronounced reduction in complex I activity have been described (Schulz et al., 2012). Moreover, studies in P301L tau mice show a downregulation of complexes I and V, accompanied by a significant reduction in MMP levels of mitochondria from 12-month-old P301L tau mice after treatment with the complex I inhibitor rotenone and the complex V inhibitor oligomycin (David et al., 2005). In addition, these animals show a decrease in complex I activity without affecting the basal respiration and the MMP which suggests a compensatory effect of other mitochondrial respiratory chain complexes. However, when the tau pathology is worsened during aging, this effect is not sufficient, and mitochondria from 24-month-old mice exhibit an impaired mitochondrial respiratory activity, diminished capacity in electron transport, and a significant reduction in ATP levels (David et al., 2005). All these events are accompanied by an increase in cytosolic H_2_O_2_ and superoxide anion radicals in 24-month-old P301L mice (David et al., 2005).

Another interesting study compares brains from mice strains with tau pathology (tripleAD; TauP301L, line pR5) in the presence (tripleAD; APP/PS2) or absence (TauP301L; WT) of Aβ production. The study demonstrates that one third of the proteins deregulated by tau pathology have functions in mitochondria and confirms differences in the expression of complex I and IV (Rhein et al., 2009). Furthermore, it shows that at 8 months, complex I activity is only decreased in pR5 mice. Interestingly, during aging, the tripleAD mice show an increase in the mitochondrial respiratory capacity compared to pR5 and APP/PS2, suggesting a synergistic effect of tau and Aβ on mitochondria (Rhein et al., 2009). Moreover, this synergistic property of Aβ seems to be related to the toxicity of different Aβ_42_ conformations. It has been shown that both oligomeric and fibril, but not monomeric Aβ_42_ cause a decrease in MMP levels in cortical neurons obtained from P301L mice (Eckert et al., 2010). In addition, mitochondrial preparations extracts from P301L mice brains show a reduction in mitochondrial respiration, respiratory control ratio, and uncoupled respiration after the treatment with oligomeric or fibril Aβ peptide (Eckert et al., 2010).

Furthermore, other tauopathies that involve an increase in tau deregulation also present mitochondrial bioenergetic dysfunction. In a segmental trisomy 16 mouse model for Down Syndrome, Ts1Cje, that presents significant tau hyperphosphorylation, decreases of ATP production and MMP as well as increases in ROS levels were shown (Esteras et al., 2017). These alterations were not related to NFT formation or APP metabolism but seem to be connected with an increase in GSK3β and JNK/SAPK activities (Shukkur et al., 2006). On the other hand, induced-pluripotent stem cells (iPSC)-derived neurons carrying the 10 + 16 MAPT mutation (inducing altered tau splicing that causes FTD) present hyperpolarization of the mitochondria, which is partially maintained by the complex V working in reverse mode, leading to an increase in ROS production, oxidative stress, and cell death (Esteras et al., 2017). Moreover, complex I respiration is inhibited, causing a decrease in the ATP production by oxidative phosphorylation that is compensated by an increase in glycolysis. Interestingly, these cells present an increase in cell death as a result of the increased rate of ROS production linked to the hyperpolarization of the mitochondria and not related with the impairment of complex I (Esteras et al., 2017).

Regarding the truncated forms of tau, our group has described that mitochondria in neuronal models expressing Asp421-cleaved tau by caspase-3 present a fragmented morphology, high levels of ROS, a significant reduction in the calcium-buffering capacity, and a significant decrease in MMP and mitochondrial membrane integrity (Quintanilla et al., 2009). Also, primary cortical neurons that express Asp421-cleaved tau enhance Aβ-induced mitochondrial failure. These observations indicate that Asp421-cleaved tau and Aβ cooperate to impair mitochondria, which likely contributes to the neuronal dysfunction in AD (Quintanilla et al., 2012). Further studies show that only aged cortical neurons expressing tau pseudo-phosphorylated at S396/404 present mitochondrial depolarization and an increase in superoxide production when these neurons are treated with Aβ (Quintanilla et al., 2014). In contrast, neurons that express Asp421-cleaved tau show a significant mitochondrial depolarization in young and aged neuronal cultures. This indicates that truncated but not phosphorylated tau may contribute to the early mitochondrial impairment reported in brain samples and neuronal cell models of AD (Quintanilla et al., 2014). Interestingly our group has described that the classic mitochondrial permeability transition pore (mPTP) inhibitor cyclosporin A (CsA) was effective in partially preventing the mitochondrial fragmentation and decrease in MMP observed in cells that express truncated tau (Quintanilla et al., 2009). Moreover, pretreatment with CsA attenuates mitochondrial membrane integrity loss after calcium overload induced by thapsigargin in immortalized cortical neurons that express Asp421-cleaved tau (Quintanilla et al., 2009).

The mPTP is a mitochondrial channel whose opening generates a non-specific increase in the permeability to ions and small solutes (Haworth and Hunter, 1979; Hunter and Haworth, 1979a,b). The original mPTP model suggests that this channel is formed by three principal proteins: cyclophilin D (CyPD), located in the mitochondrial matrix; the adenine nucleotide translocator (ANT), found in the inner membrane; and the voltage-dependent anion channel (VDAC) in the outer membrane (Rao et al., 2014). However, recently it was proposed that ATP synthase is also a major component of the mPTP, for review see Perez et al., 2017). In the most common neurological disorders, the impairment of the calcium regulation and increased ROS levels are potent inducers of an mPTP opening (Du and Yan, 2010). The formation and the consequent opening of the mPTP is a key factor in mitochondrial dysfunction and mitochondria-driven cell death, as this process involves a failure in MMP, a decrease in ATP production, release of mitochondrial content, and finally cell death (Kroemer and Blomgren, 2007; Bonora et al., 2013; Bernardi and Di Lisa, 2015; Bernardi et al., 2015; Jonas et al., 2015).

Interestingly, Asp421-cleaved tau is not the only pathological form that affects mitochondria through the mPTP opening. Amadoro and colleagues overexpressed some N-terminal derived fragments of tau located around different protease(s)-cleavage consensus sites in the tau NH2 domain. They show that tau N-terminal fragments lacking the first 25 amino acids induce neurodegeneration, cell death, and synaptic failure (Amadoro et al., 2006). More importantly, this NH2-26-44 tau fragment can impair oxidative phosphorylation due to the non-competitive inhibition of the mitochondrial ANT, an ADP/ATP exchanger (Atlante et al., 2008). In addition, Amadoro and colleagues found that the NH2-derived tau fragment preferentially interacts with Aβ peptide in human AD tissues in association with mitochondrial ANT and CypD. These interactions between the tau fragments and Aβ exacerbate the ANT impairment, thereby potentiating ANT dysfunction and further decrease the ATP production (Amadoro et al., 2012). Moreover, they describe that the addition of the VDAC inhibitor DIDS reduces the levels of the mitochondrial superoxide anions produced in these cells, which is caused by a dysfunctional complex I activity (Amadoro et al., 2012). Finally, superoxide levels increases, that are generated by the presence of this tau fragment, modify the active site of mitochondrial ANT, thereby directly influencing the opening of the mPTP (Bobba et al., 2013). On the other hand, the mPTP component VDAC was also analyzed in postmortem brains of AD patients and brain samples from APP, APP/PS1, and tripleAD mice (Manczak and Reddy, 2012b). These studies show progressively increased levels of VDAC in the cortical tissues from AD brains compared to control subjects (Manczak and Reddy, 2012b). It was also found that VDAC1 interacts with Aβ and phosphorylated tau in the brain of these AD mice models suggesting that this interaction may block mitochondrial pores, leading to defects in oxidative phosphorylation and mitochondrial dysfunction (Manczak and Reddy, 2012b). Although several proteins and bioenergetics deregulation mediated by tau pathology could be associated with the opening of the mPTP (**Figure 3**), further investigation is necessary to unravel this pending issue.

## Final Remarks

Even under normal physiological conditions, tau can undergo different posttranslational modifications that play various roles in the onset and progression of AD. Several of these modifications may have converging mechanisms of toxicity, but to date, it is unknown how these posttranslational modifications affect neuronal function and trigger different pathological conditions. Here, we showed that the presence of tau pathology could affect mitochondria function, which it seems to explain better the neuronal dysfunction observed in AD. We described evidence indicating that the presence of pathological forms of tau negatively affects the mitochondria trafficking, morphology, and bioenergetics. These actions compromise mitochondrial function generating: (i) a decrease in local ATP levels, which impairs normal neurotransmission, and (ii) an imbalance in the calcium buffering capacity with subsequent neurotoxicity. Both mechanisms can directly affect the neuronal metabolism and further brain functions. While many questions remain, a better understanding of the early events in tau-mediated neurotoxicity is particularly important as it may lead to the development of new therapeutic strategies that prevent the impairment of mitochondrial function and eventually decrease the pathological neuronal events that initiate neurodegeneration.

## Author Contributions

MP performed the research, wrote the paper, drew the figures, and revised the paper. CJ performed the research and wrote the paper. RQ directed the project, supported the research, and revised the paper.

## Conflict of Interest Statement

The authors declare that the research was conducted in the absence of any commercial or financial relationships that could be construed as a potential conflict of interest.
